# Supplementary material for: Occupancy of wild southern pig-tailed macaques in intact and degraded forests in Peninsular Malaysia
Source: PeerJ. 2021 Dec 14;9:e12462. doi: 10.7717/peerj.12462 (PMC8679909; doi:10.7717/peerj.12462)
Supplement: Supplemental Information 1 [file peerj-09-12462-s001.pdf]

Occupancy of wild southern pig-tailed macaques in intact and degraded forests in Peninsular Malaysia

Anna Holzner, D. Mark Rayan, Jonathan Moore, Cedric Kai Wei Tan, Laura Clart, Lars Kulik, Hjalmar Kühl, Nadine Ruppert and Anja Widdig

Supplemental Tables

Table S1. Candidate models for the detection of *Macaca nemestrina* in the Pasoh Forest Reserve (PFR) and Belum-Temengor Forest Complex (BTFC) with global occupancy models. Shown are Akaike's Information Criterion corrected for small samples (AIC_c), differences in AIC_c between each model and the respective best model (ΔAIC_c), the probability of each model to be the best model, i.e., the Akaike weights (wAIC), and the number of parameters (K). Top-ranked models ($\Delta AIC_c \leq 2$) are indicated in bold.

Candidate models	AIC_c	ΔAIC_c	wAIC	K
PFR, ψ (edge + elevation) γ (forest degradation) ε (forest degradation)				
***p* (effort + sampling year)**	**2069.5**	**0**	**1**	**15**
p (effort)	2092.5	22.96	0	11
p (effort + sampling year + sampling month)	2094.4	24.90	0	24
p (effort + sampling month)	2106.7	37.25	0	20
p (sampling year)	2143.5	74.01	0	14
p (.)	2162.8	93.27	0	10
p (sampling year + sampling month)	2162.9	93.42	0	23
p (sampling month)	2175.1	105.65	0	19
BTFC, ψ (habitat + NDVI + settlement + elevation)				
***p* (effort + sampling month)**	**1238.6**	**0**	**0.995**	**16**
p (effort)	1249.4	10.77	0.005	7
p (sampling month)	1270.8	32.21	0	15
p (.)	1283.0	44.38	0	6

Table S2. Effect of covariates on the detection of *Macaca nemestrina* in the Pasoh Forest Reserve (PFR) and Belum-Temengor Forest Complex (BTFC). Shown are model averaged estimates (zero method), standard errors (SE), and lower and upper 95% confidence intervals (CI). Predictors included into the respective top models ($\Delta AIC_c \leq 2$) are indicated in bold.

Site	Parameter	Covariate	Estimate	SE	lower CI	upper CI
PFR	Detection *p*	**effort[a]**	**0.51**	**0.06**	**0.39**	**0.63**
		**sampling year (2013 vs. 2014)[b]**	**-0.70**	**0.18**	**-1.05**	**-0.34**
		**sampling year (2013 vs. 2015)[b]**	**-0.96**	**0.18**	**-1.32**	**-0.61**
		**sampling year (2013 vs. 2016)[b]**	**-0.71**	**0.18**	**-1.05**	**-0.36**
		**sampling year (2013 vs. 2017)[b]**	**-0.23**	**0.19**	**-0.60**	**0.14**
		sampling month (Jan vs. Feb)[c]	0.000009	0.005	-0.29	4.64
		sampling month (Jan vs. May)[c]	0.000004	0.002	-0.03	2.23
		sampling month (Jan vs. Jun)[c]	0.000004	0.002	-0.04	2.18
		sampling month (Jan vs. Jul)[c]	0.000004	0.002	-0.23	2.02
		sampling month (Jan vs. Aug)[c]	0.000002	0.002	-0.61	1.75
		sampling month (Jan vs. Sep)[c]	0.000006	0.003	0.11	3.03
		sampling month (Jan vs. Oct)[c]	0.000005	0.003	0.08	2.45
		sampling month (Jan vs. Nov)[c]	0.000002	0.002	-0.68	1.69
		sampling month (Jan vs. Dec)[c]	0.000002	0.002	-0.61	1.86
BTFC	Detection *p*	**effort[a]**	**0.61**	**0.12**	**0.37**	**0.84**
		**sampling month (Jan vs. Feb)[c]**	**0.25**	**0.26**	**-0.25**	**0.76**
		**sampling month (Jan vs. Mar)[c]**	**0.12**	**0.27**	**-0.40**	**0.64**
		**sampling month (Jan vs. Apr)[c]**	**0.44**	**0.32**	**-0.19**	**1.08**
		**sampling month (Jan vs. May)[c]**	**0.99**	**0.54**	**-0.06**	**2.04**
		**sampling month (Jan vs. Aug)[c]**	**-11.12**	**314.10**	**-628.20**	**605.87**
		**sampling month (Jan vs. Sep)[c]**	**-2.07**	**1.05**	**-4.12**	**-0.03**
		**sampling month (Jan vs. Oct)[c]**	**-0.80**	**0.34**	**-1.45**	**-0.15**
		**sampling month (Jan vs. Nov)[c]**	**-0.46**	**0.30**	**-1.04**	**0.12**
		**sampling month (Jan vs. Dec)[c]**	**-0.37**	**0.30**	**-0.96**	**0.22**

[a] z-transformed to mean = 0 and SD = 1 prior to model fitting; original means ± SDs were: effort (*PFR*): 6.0 ± 1.8 days, effort (*BTFC*): 12.1 ± 3.6 days.
[b] Reference level is '2013'.
[c] Reference level is 'Jan'.

Table S3. Candidate models for *Macaca nemestrina* occupancy in the Pasoh Forest Reserve (PFR) and Belum-Temengor Forest Complex (BTFC) with best respective detection models. Shown are Akaike's Information Criterion corrected for small samples (AIC_c), differences in AIC_c between each model and the respective best model (ΔAIC_c), the probability of each model to be the best model, i.e., the Akaike weights (wAIC), and the number of parameters (K). Top-ranked models ($\Delta AIC_c \leq 2$) are indicated in bold.

Candidate models	AIC_c	ΔAIC_c	wAIC	K
PFR, *p* (effort + sampling year)				
**ψ (.) γ (.) ε (forest degradation)**	**2056.4**	**0**	**0.633**	**11**
ψ (elevation) γ (.) ε (forest degradation)	2059.4	3.03	0.139	12
ψ (edge) γ (.) ε (forest degradation)	2059.5	3.05	0.138	12
ψ (.) γ (forest degradation) ε (forest degradation)	2062.6	6.21	0.028	13
ψ (edge + elevation) γ (.) ε (forest degradation)	2062.7	6.30	0.027	13
ψ (.) γ (.) ε (.)	2064.0	7.60	0.014	9
ψ (elevation) γ (forest degradation) ε (forest degradation)	2065.9	9.52	0.005	14
ψ (edge) γ (forest degradation) ε (forest degradation)	2065.9	9.54	0.005	14
ψ (elevation) γ (.) ε (.)	2066.8	10.38	0.004	10
ψ (edge) γ (.) ε (.)	2066.8	10.40	0.003	10
ψ (edge + elevation) γ (forest degradation) ε (forest degradation)	2069.5	13.09	0.001	15
ψ (.) γ (forest degradation) ε (.)	2069.7	13.29	0.001	11
ψ (edge + elevation) γ (.) ε (.)	2069.8	13.39	0.001	11
ψ (elevation) γ (forest degradation) ε (.)	2072.7	16.32	0	12
ψ (edge) γ (forest degradation) ε (.)	2072.7	16.33	0	12
ψ (edge + elevation) γ (forest degradation) ε (.)	2076.0	19.59	0	13
BTFC, *p* (effort + sampling month)				
**ψ (elevation)**	**1232.1**	**0**	**0.415**	**13**
ψ (NDVI + elevation)	1234.2	2.04	0.149	14
ψ (settlement + elevation)	1234.3	2.19	0.139	14
ψ (habitat + elevation)	1234.4	2.24	0.135	14
ψ (NDVI + settlement + elevation)	1236.3	4.19	0.051	15
ψ (habitat + NDVI + elevation)	1236.4	4.28	0.049	15
ψ (habitat + settlement + elevation)	1236.6	4.42	0.046	15
ψ (habitat + NDVI + settlement + elevation)	1238.6	6.45	0.016	16
ψ (habitat + settlement)	1260.9	28.78	0	14
ψ (habitat + NDVI + settlement)	1261.2	29.07	0	15
ψ (habitat)	1268.8	36.66	0	13
ψ (habitat + NDVI)	1269.4	37.27	0	14
ψ (NDVI + settlement)	1271.4	39.29	0	14
ψ (NDVI)	1271.7	39.54	0	13
ψ (.)	1274.3	42.19	0	12
ψ (settlement)	1275.6	43.51	0	13
