# Supplementary material for: Occupancy of wild southern pig-tailed macaques in intact and degraded forests in Peninsular Malaysia
Source: PeerJ. 2021 Dec 14;9:e12462. doi: 10.7717/peerj.12462 (PMC8679909; doi:10.7717/peerj.12462)
Supplement: Supplemental Information 2 [file peerj-09-12462-s002.pdf]

Occupancy of wild southern pig-tailed macaques in intact and degraded forests in Peninsular Malaysia

Anna Holzner, D. Mark Rayan, Jonathan Moore, Cedric Kai Wei Tan, Laura Clart, Lars Kulik, Hjalmar Kühl, Nadine Ruppert and Anja Widdig

Supplemental Methods

Occupancy modelling

MacKenzie et al. (2006) described occupancy as the probability with which a species occurs at a specific site. Yet, during wildlife surveys, individuals often remain undetected though being present, which may ultimately cause underestimation of site occupancy or biases in estimates of local colonization and extinction (MacKenzie et al., 2003). Animals' detection may further vary over time, due to environmental factors, or as a result of observational differences. Occupancy models account for imperfect and varying detection probabilities by linking a state model determining occupancy (ψ) with an observation model determining detection (p, MacKenzie et al., 2002).

Statistical Analysis

Occupancy analyses were implemented using the package 'unmarked' (version 1.0.1, Fiske & Chandler, 2011) in R (version 3.4.4, R Core Team, 2018). Dynamic and single-season occupancy models for PFR and BTFC, respectively, were fitted using the functions *colext* and *occu* from this package. Candidate models based on all possible combinations of predictor sets were built using the function *dredge* from the package 'MuMIn' (version 1.43.17, Barton, 2020). Model averaged estimates were obtained using the function *model.avg* from the package 'MuMIn'. The goodness of fit of the global models was tested using the function *mb.gof.test* from the package 'AICcmodavg' (version 2.3.1, Mazerolle, 2020).

Note that the functions *colext* and *occu* produce estimates on the logit scale (Fiske & Chandler, 2011). In order to back-transform them to the original scale, we used the function *predict* of the package 'unmarked'. Further, dynamic occupancy models provide estimates only for first-year occupancy. Predictions for subsequent years were derived from the two parameters governing the dynamics, i.e., extinction and colonization, using the function *smoothed* from the package 'unmarked'. Unlike the projected trajectory that precisely follows the model, the smoothed trajectory is informed by both the model as well as the sample data, thus having been suggested to provide a better representation of the true pattern of occupancy dynamics (Weir, Fiske & Royle, 2009).

Barton K. 2020. Mu-MIn: Multi-model inference. Available at https://CRAN.R-project.org/package=MuMIn (accessed 24 February 2021).

Fiske I, Chandler R. 2011. unmarked: An R package for fitting hierarchical models of wildlife occurrence and abundance. *Journal of Statistical Software* 43:1–23. DOI: 10.18637/jss.v043.i10.

Kery M, Chandler R. 2016. Dynamic occupancy models in unmarked. *Swiss Ornithological Institute and University of Georgia*. Available at https://cran.r-project.org/web/packages/unmarked/vignettes/colext.pdf (accessed 24 February 2021).

MacKenzie DI, Nichols JD, Hines JE, Knutson MG, Franklin AB. 2003. Estimating site occupancy, colonization, and local extinction when a species is detected imperfectly. *Ecology* 84:2200–2207. DOI: 10.1890/02-3090.

MacKenzie DI, Nichols JD, Lachman GB, Droege S, Royle JA, Langtimm CA. 2002. Estimating site occupancy rates when detection probabilities are less than one. *Ecology* 83:2248–2255. DOI: 10.1890/0012-9658(2002)083[2248:ESORWD]2.0.CO;2.

MacKenzie DI, Nichols JD, Royle JA, Pollock KH, Bailey LL, Hines JE. 2006. *Occupancy Estimation and Modeling: Inferring Patterns and Dynamics of Species Occurrence*. Burlington, MA: Elsevier Academic Press.

Mazerolle MJ. 2020. Model selection and multimodel inference using the AICcmodavg package. Available at https://cran.r-project.org/package=AICcmodavg (accessed 24 February 2021).

R Core Team. 2018. *R: A language and environment for statistical computing.* Vienna, Austria: R Foundation for Statistical Computing. Available at https://www.R-project.org (accessed 24 February 2021).

Weir L, Fiske IJ, Royle JA. 2009. Trends in anuran occupancy from northeastern states of the North American Monitoring Program. *Herpetological Conservation and Biology* 4:389–402.
